# Monitoring Enteroviruses and SARS-CoV-2 in Wastewater Using the Polio Environmental Surveillance System in Japan

**DOI:** 10.1128/aem.01853-22

**Published:** 2023-03-28

**Authors:** Kazuhiro Kitakawa, Kouichi Kitamura, Hiromu Yoshida

**Affiliations:** a Department of Microbiology, Fukushima Prefectural Institute of Public Health, Fukushima, Japan; b Department of Virology II, National Institute of Infectious Diseases, Tokyo, Japan; University of Nebraska—Lincoln

**Keywords:** environmental surveillance, poliovirus, enterovirus, SARS-CoV-2, pepper mild mottle virus

## Abstract

In the global strategy for polio eradication, environmental surveillance (ES) has been established worldwide to monitor polioviruses. In addition, nonpolio enteroviruses are simultaneously isolated from wastewater under this ES program. Hence, ES can be used to monitor enteroviruses in sewage to supplement clinical surveillance. In response to the coronavirus disease 2019 (COVID-19) pandemic, we also monitored severe acute respiratory syndrome coronavirus 2 (SARS-CoV-2) in sewage using the polio ES system in Japan. Enterovirus and SARS-CoV-2 were detected in sewage from January 2019 to December 2021 and from August 2020 to November 2021, respectively. Enterovirus species such as echoviruses and coxsackieviruses were frequently detected by ES in 2019, indicating the circulation of these viruses. After the onset of the COVID-19 pandemic, sewage enterovirus detection and related patient reports were notably reduced in 2020 to 2021, suggesting changes in the hygiene behaviors of the human population in response to the pandemic. Our comparative experiment with a total of 520 reverse transcription-quantitative PCR (RT-qPCR) assays for SARS-CoV-2 detection demonstrated that the solid-based method had a significantly higher detection rate than that of the liquid-based method (24.6% and 15.9%, respectively). Moreover, the resulting RNA concentrations were correlated with the number of new COVID-19 cases (Spearman’s *r* = 0.61). These findings indicate that the existing polio ES system can be effectively used for enterovirus and SARS-CoV-2 sewage monitoring using different procedures such as virus isolation and molecular-based detection.

**IMPORTANCE** Long-term efforts are required to implement surveillance programs for the ongoing COVID-19 pandemic, and they will be required even in the postpandemic era. We adopted the existing polio environmental surveillance (ES) system for SARS-CoV-2 sewage monitoring in Japan as a practical and cost-effective approach. Moreover, the ES system routinely detects enteroviruses from wastewater and, therefore, can be used for enterovirus monitoring. The liquid fraction of the sewage sample is used for poliovirus and enterovirus detection, and the solid fraction can be used for SARS-CoV-2 RNA detection. The present study demonstrates how the existing ES system can be used for monitoring enteroviruses and SARS-CoV-2 in sewage.

## INTRODUCTION

In 1988, the Global Polio Eradication Initiative (GPEI) was launched by the World Health Organization (WHO), and in 2020, five of the six WHO regions were certified as wild-polio free; these include the Americas, the Western Pacific, Europe, Southeast Asia, and Africa (1–4). To maintain a polio-free status, environmental surveillance (ES), in addition to clinical surveillance (CS), has been implemented in many countries with a risk of the emergence/reemergence of polio (5, 6). Polio ES was pioneered in Japan (7, 8), and national polio ES has been performed routinely since 2013 (9). The polio ES network in Japan consists of the National Institute of Infectious Diseases (NIID) and prefectural public health institutes (PHIs) based on the National Epidemiological Surveillance of Vaccine-Preventable Diseases (NESVPD) program. In the prefectures participating in this ES program, influent wastewater samples are collected monthly from different wastewater treatment plants (WWTPs) and analyzed by PHIs. If poliovirus is isolated from a sample at a PHI, the sample is shipped to the national polio laboratory at the NIID for further diagnosis. Since the introduction of the inactivated poliovirus vaccine for routine immunization and the achievement of a polio-free status in Japan, nonpolio enteroviruses (NPEVs), adenoviruses, and reoviruses are often detected in wastewater using this surveillance program (10, 11). Furthermore, these secondary results might be used to comprehensively trace the circulation of enteroviruses in the human population (11–14). In Japan, the results of virus isolation are reported annually as a part of NESVPD reports.

After the onset of the coronavirus disease 2019 (COVID-19) pandemic in 2020, wastewater surveillance of severe acute respiratory syndrome coronavirus 2 (SARS-CoV-2) emerged as an effective approach to complement CS (15). Numerous studies have reported the detection of SARS-CoV-2 RNA in wastewater, and some have demonstrated an association between the RNA concentrations detected in wastewater and the number of reported COVID-19 cases in the community (16–20). Similar to enterovirus monitoring, the national polio ES network can likely be used for monitoring sewage SARS-CoV-2 as a research project (21, 22). While poliovirus and enterovirus are commonly detected by virus isolation tests, sewage SARS-CoV-2 is detected directly using reverse transcription-quantitative PCR (RT-qPCR) assays. Various factors, including the virus decay rate, temperature, and population, cause uncertainties in estimations of prevalence from sewage SARS-CoV-2 RNA concentrations (23). Virus recovery from wastewater using concentration methods has been developed primarily for nonenveloped viruses such as poliovirus and norovirus rather than for enveloped coronaviruses (24). The WHO guideline for polio ES recommends the two-phase separation method using the liquid fraction of wastewater (25). Other WHO-accepted methods such as electronegative-membrane adsorption and polyethylene glycol (PEG) precipitation also use the liquid fraction (26). Although the rate of detection of the presence of SARS-CoV-2 RNA in wastewater is very high in prevalent areas, the level of virus excretion per infected person is several orders of magnitude lower for SARS-CoV-2 than for poliovirus or norovirus (27), and hence, its detection requires a novel, sensitive method. Recent studies analyzed solid fractions that were precipitated after the low-speed centrifugation of wastewater to monitor SARS-CoV-2 (28–34). Our previous study revealed the considerably more efficient detection of SARS-CoV-2 RNA in the solid fraction than in the liquid fraction via the negative-membrane adsorption method (31). Similarly, Peccia et al. detected a high concentration of SARS-CoV-2 RNA in the sludge of a WWTP (35). It is reasonable to assume that the solid fraction discarded from routine polio ES can be used to monitor sewage SARS-CoV-2.

In this study, we aimed to use the existing national polio ES system in Japan to monitor both enteroviruses and SARS-CoV-2 in wastewater. The frequency of enterovirus isolates between January 2019 and December 2021 and the concentrations of SARS-CoV-2 RNA between August 2020 and November 2021 were monitored. Using a total of 81 raw sewage samples collected from two WWTPs, we performed a systematic comparison of solid- and liquid-based methods to evaluate the usefulness of the solid fraction for SARS-CoV-2 RNA detection. This study provides insights into the practical applications of the national polio ES network for monitoring enteroviruses and coronaviruses.

## RESULTS

### Enterovirus detection at polio ES sites.

**FIG 1:**
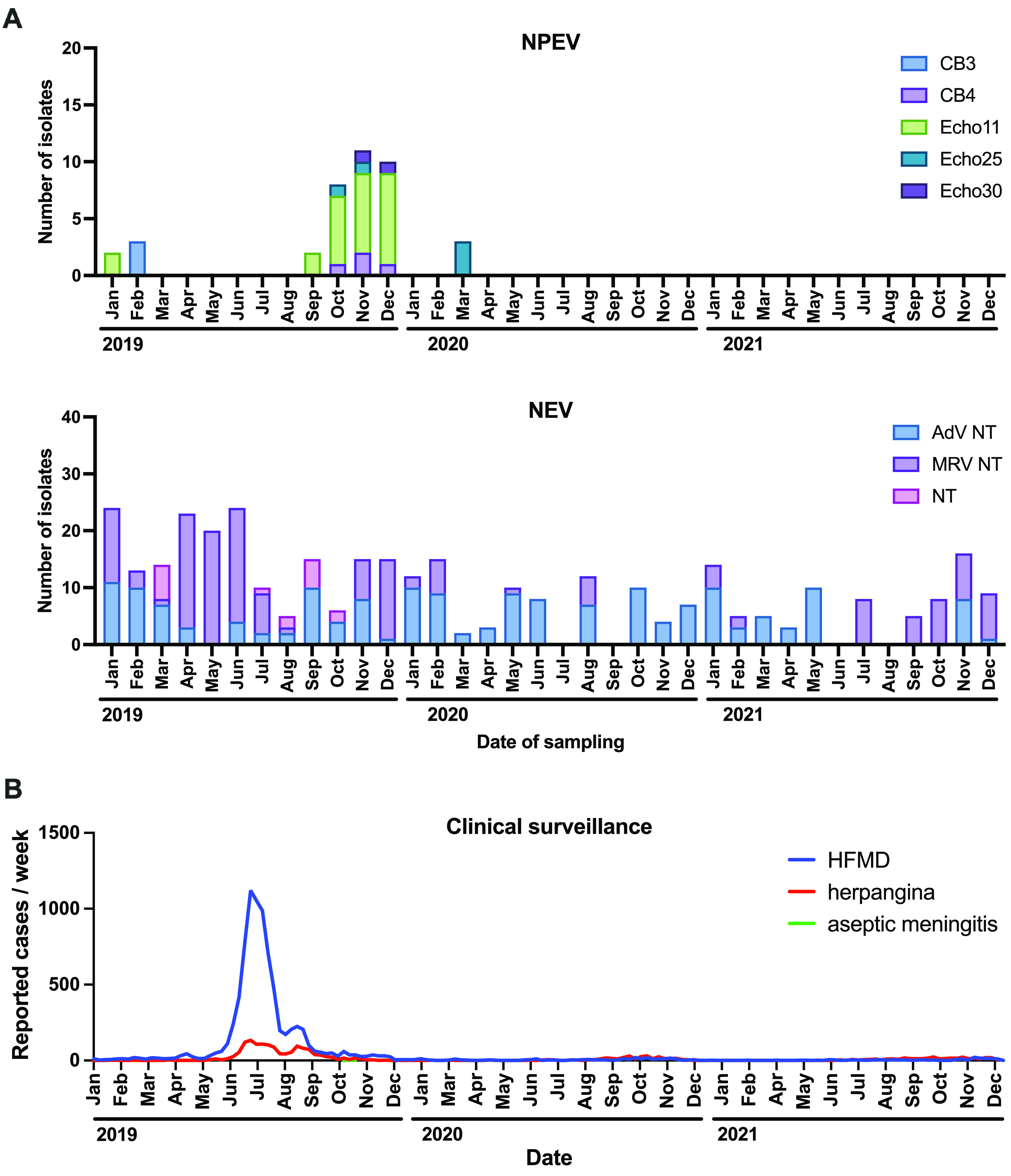
Summary of the frequencies of virus isolation from wastewater and the reported numbers of cases associated with enterovirus in sentinel sites of an anonymous prefecture in Japan from 2019 to 2021. (A) Frequencies of isolation of nonpolio enteroviruses (NPEV) with serotypes and nonenterovirus (NEV). (B) Graph of clinical surveillance (pediatric sentinel surveillance) showing enterovirus-associated cases of hand-foot-and-mouth disease (HFMD), herpangina, and aseptic meningitis. Adenovirus (AdV) and mammalian orthoreovirus (MRV) were not typed (NT). No poliovirus was isolated. CB, coxsackievirus B; Echo, echovirus.

Routine polio ES is performed on a monthly basis with samples collected from WWTP-A (Table 1). A total of 373 cytopathic effect (CPE) agents from 1,440 wells were obtained from 36 sewage samples during the period from January 2019 to December 2021 (see Materials and Methods for details). The results of virus identification and molecular typing are summarized in Fig. 1A. Coxsackievirus B3 (CB3), CB4, echovirus 11 (Echo11), Echo25, and Echo30 were isolated as NPEVs, and Echo11 showed the highest frequency. Notably, no NPEVs were isolated between April 2020 and December 2021. Figure 1B shows the CS data for pediatric sentinel sites in the same prefecture (https://www.pref.fukushima.lg.jp/sec/21910a/kansenshojoho.html) where WWTP-A is located. Similar to the ES results, the frequency of the occurrence of enterovirus-related diseases such as hand-foot-and-mouth disease (HFMD), herpangina, and aseptic meningitis was substantially lower in 2020 to 2021 than in 2019. In contrast, adenovirus (AdV) and reovirus (mammalian orthoreovirus [MRV]) were constantly isolated as nonenteroviruses (NEVs) during the 2019–2021 sampling period.

**TABLE 1:** Sampling site characteristics^*a*^

WWTP	Catchment area population	No. of establishments
A	~100,000–200,000	18,000
B	~200,000–500,000	25,000

a Medical institutions designated for infectious diseases were available in both areas. WWTP, wastewater treatment plant.

### SARS-CoV-2 detection at the polio ES sites.

**FIG 2:**
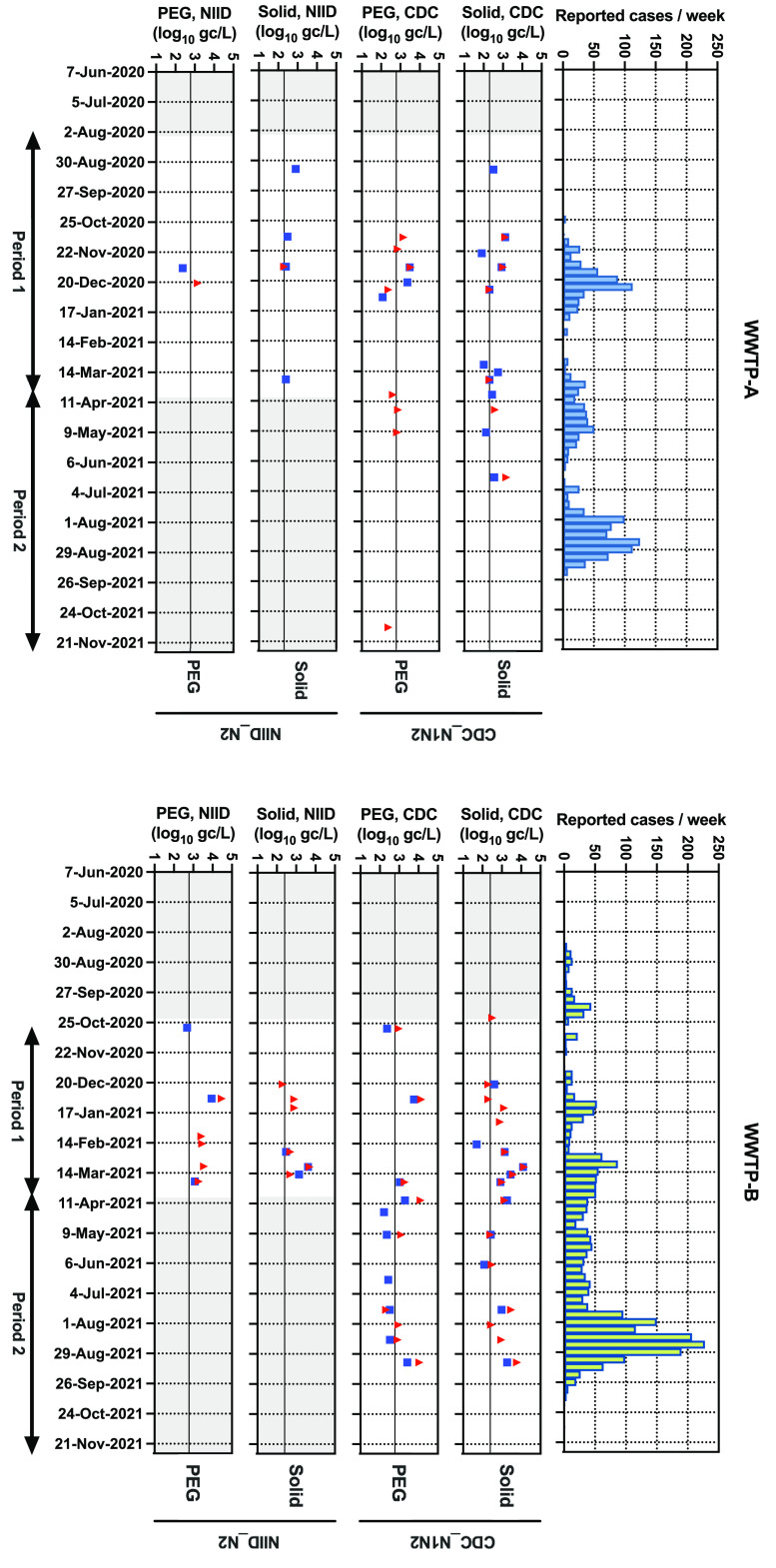
Summary of the numbers of new COVID-19 cases and SARS-CoV-2 RNA concentrations in wastewater samples obtained from WWTP-A and WWTP-B. The numbers of new COVID-19 cases per week in each catchment area are included. Data on RNA concentrations are presented separately based on the virus recovery method (solid based and PEG precipitation) and primer/probe sets (CDC N1/N2 and NIID N2). The blue squares and red triangles indicate results from the 1-step and 2-step RT-qPCR procedures, respectively. The limits of quantification (LOQs) for wastewater based on RT-qPCR assays are as follows: 200 gc/L for solid and 600 gc/L for PEG in WWTP-A and 250 gc/L for solid and 600 gc/L for PEG in WWTP-B.

WWTP-B was added as a sampling site to monitor SARS-CoV-2. A total of 81 samples from two WWTPs were analyzed in parallel using different virus recovery methods (solid based and PEG precipitation), primer/probe sets (NIID N2 and CDC N1/N2), and RT-qPCR procedures (one step and two steps). The results from 520 RT-qPCR assays for the entire monitoring period are summarized as the calculated gene copies (gc) per liter of wastewater with new COVID-19 cases reported in the catchment areas of both WWTPs (Fig. 2). The RNA concentrations in the positive samples (98 positive samples/520 assays) are presented separately according to the methods used. Based on the interim results from the relatively lower-sensitivity NIID N2 assay during period 1 (August 2020 to March 2021), monitoring using the NIID N2 assay ceased at the end of March 2021, and the CDC N1/N2 assay alone was used during period 2 (April 2021 to November 2021) for both WWTPs. In the CDC N1/N2 assay, positive signals were detected at approximately the same time as when multiple surges of new COVID-19 cases were detected in both WWTPs. In WWTP-A, between July 2021 and September 2021, there were no positive signals in the wastewater, while a surge in new cases was observed. A notable reduction in the pepper mild mottle virus (PMMoV) RNA concentration was not observed for the same wastewater samples (see Fig. S1 in the supplemental material).

### Comparison of virus recovery and RT-qPCR methods.

**FIG 3:**
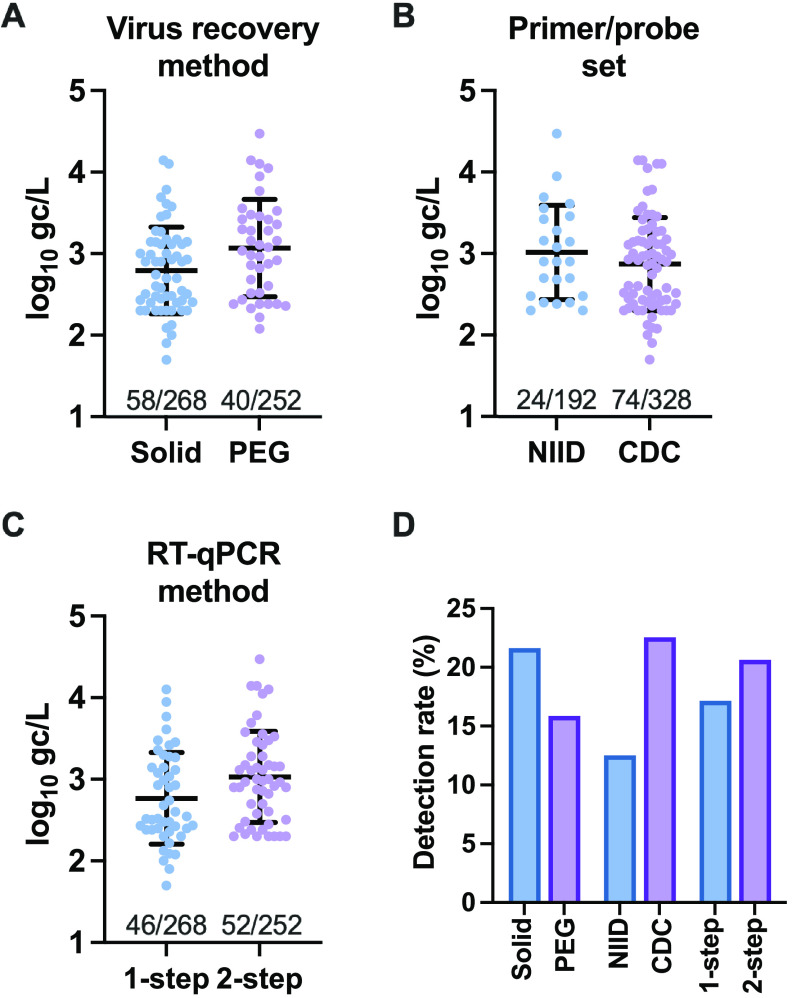
(A to C) Scatterplots representing the RNA concentrations in wastewater samples (log_10_ gene copies [gc] per liter). Virus recovery methods (solid based and PEG precipitation) (A), primer/probe sets (CDC N1/N2 and NIID N2) (B), and RT-qPCR procedures (1 step and 2 steps) (C) were compared for 520 assays. Geometric means and geometric standard deviations are presented. Values at the bottom of the graphs represent the ratio of the number of positive samples to the total number of analyzed samples. (D) Detection rates for each method (solid based and PEG precipitation), primer/probe set (CDC N1/N2 and NIID N2), and RT-qPCR procedure (1 step and 2 steps).

Figure 3 summarizes the results of 520 SARS-CoV-2 RT-qPCR assays. PEG precipitation yielded a higher geometric mean SARS-CoV-2 RNA concentration (1,174 gc/L) than the solid-based method (621 gc/L); however, the detection rate of the solid-based method (58 positive/268 total samples; 24.6%) was higher than that of PEG precipitation (40/252; 15.9%). The CDC N1/N2 duplex assay showed a higher detection rate (74/328; 22.6%) than that of the NIID N2 assay (24/192; 12.5%). Two-step RT-qPCR yielded a slightly higher detection rate (20.6%) than that of the one-step assay (17.2%). The CDC N1/N2 assay showed a significantly higher sensitivity than the NIID N2 assay (*P* < 0.005) (Table 2), as suggested by the results of McNemar’s test. Among the conflicting results, four samples were positive based on the results of the NIID N2 assay alone. The solid-based method showed a relatively higher detection sensitivity, which was significant (*P* < 0.05). RT-qPCR did not reveal a significant difference between the one-step and two-step procedures.

**TABLE 2:** Results of McNemar’s test^*a*^

Test and result	No. of samples with result			*P* value
	−	+	Total	
CDC N1/N2	NIID N2			
−	147	4	151	0.0022
+	20	20	40	
Total	167	24	191	
Solid	PEG			
−	170	19	189	0.0310
+	36	21	57	
Total	206	40	246	
2-step RT-qPCR	1-step RT-qPCR			
−	179	13	192	0.1763
+	22	30	52	
Total	201	43	244	

a Solid, solid-fraction-based method; PEG, polyethylene glycol precipitation; NIID N2, National Institute of Infectious Diseases nucleocapsid assay; CDC N1/N2, Centers for Disease Control and Prevention nucleocapsid assay.

### Internal control for wastewater samples.

**FIG 4:**
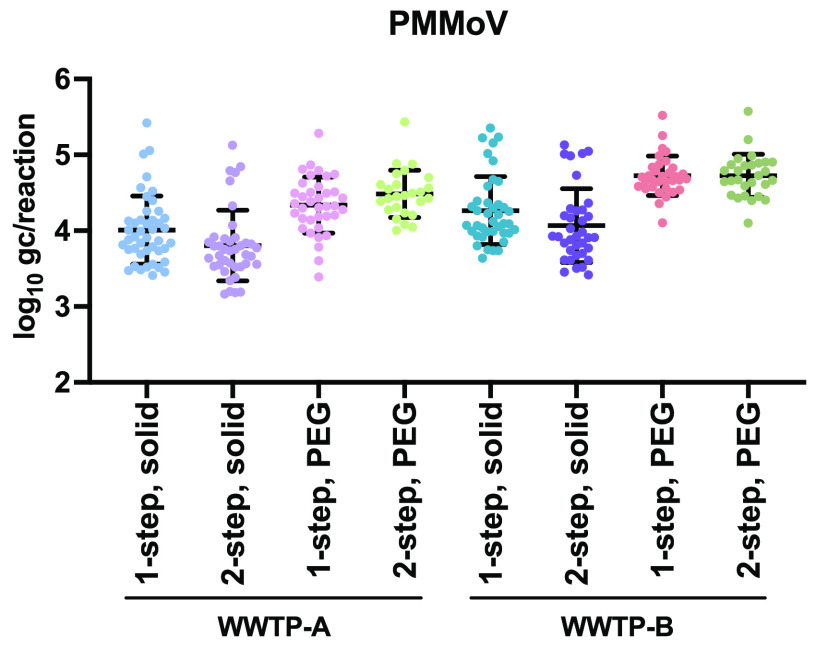
Scatterplots representing the concentrations of PMMoV RNA as an internal control in wastewater samples in log_10_ gene copies (gc) per well by RT-qPCR assays. All tested RNA (1-step) and cDNA (2-step) samples examined using the SARS-CoV-2 assays were subjected to parallel analysis using the PMMoV assays. Geometric means and geometric standard deviations are presented.

The plant virus PMMoV is very abundant in wastewater and is used as an internal control for virus recovery, RNA extraction, and RT-qPCR (31, 36–39). We found high concentrations of PMMoV RNA in all of the tested samples (Fig. 4), validating the whole process of detection. As a result, no detrimental effects that could be attributed to PCR inhibitors present in wastewater were observed. The PMMoV RNA concentration in the solid fraction was approximately 10-fold lower than that in the liquid fraction of wastewater samples derived from both WWTPs, suggesting that the partition trend of PMMoV prefers the liquid fraction.

### Association between the sewage SARS-CoV-2 RNA concentrations and the numbers of new COVID-19 cases in catchment areas.

**FIG 5:**
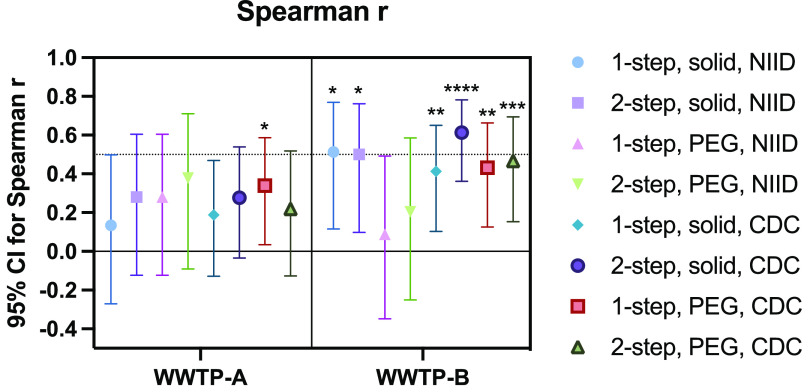
Correlation between the reported number of new COVID-19 cases and SARS-CoV-2 RNA concentrations, analyzed using Spearman’s rank correlation test. Spearman’s *r* values are presented. *, *P* < 0.05; **, *P* < 0.01; ***, *P* < 0.005; ****, *P* < 0.0001. CI, confidence interval.

The number of COVID-19 cases was significantly correlated with the viral RNA concentrations determined via the “PEG, CDC N1/N2, 1-step” method using samples from WWTP-A (*r* = 0.34; *P* < 0.05) (Fig. 5), as suggested by the results of Spearman’s correlation analysis; however, only three samples showed a positive correlation (Fig. 3). Analysis of samples from WWTP-B indicated a relatively higher correlation coefficient for CDC N1/N2 assays, especially the “2-step, solid” (*r* = 0.61; *P* < 0.0001) and “2-step, PEG” (*r* = 0.47; *P* < 0.005) methods (Fig. 5). Taken together, both virus recovery methods combined with the CDC N1/N2 RT-qPCR assay showed efficient detection that was significantly correlated with new COVID-19 cases in the catchment areas of large WWTPs.

## DISCUSSION

This study demonstrated the results of enterovirus and SARS-CoV-2 monitoring using the established polio ES system in Japan. Additionally, methods for detecting SARS-CoV-2 RNA in sewage, namely, the PEG precipitation method and the solid-based method, were systematically compared. Although enteroviruses, including poliovirus, have been previously isolated using the liquid and solid fractions of wastewater (40), standard polio ES protocols use the liquid fraction for different methods such as the two-phase method (WHO protocol) (25) and the negative-membrane adsorption method (12). Thus, we tested only the liquid fraction for enterovirus isolation. In the present study, Echo11 isolates were frequently identified during autumn and winter (Fig. 1A). Even though Echo11 is one of the major pathogens of aseptic meningitis, CS of aseptic meningitis and both herpangina and aseptic meningitis at pediatric sentinel sites in the same prefecture showed a low correlation with ES in 2019 (Fig. 1B). A similar trend of a peak lag was previously reported for another prefecture in Japan (11). As the clinical data in the present study were obtained from pediatric sentinel surveillance, asymptomatic infections among adults and children could have resulted in these peak lags. In contrast, the striking reductions in the frequencies of sewage enterovirus isolates and enterovirus-associated diseases in 2020 to 2021 (Fig. 1) clearly indicated the correlation between ES and pediatric sentinel surveillance. This massive reduction suggests that the COVID-19 pandemic drastically changed people’s hygiene behaviors, such as the frequency of handwashing. However, the reason behind the constant detection of adenovirus and reovirus is not known (Fig. 1B). In cases of virus coinfection during virus isolation tests, the presence of enterovirus can interfere with adenovirus replication (41). The occasion of adenovirus isolation might have increased between 2020 and 2021. Furthermore, reovirus has a wide range of hosts, and hence, humans are not the only source of sewage reovirus (42).

For sewage SARS-CoV-2 monitoring, the performance of the solid-based method was significantly better than that of the liquid-based (PEG precipitation) method, as indicated by the results of McNemar’s test (Table 2). However, Spearman’s test indicated that both methods showed a significant correlation between the RNA concentration and the number of reported COVID-19 cases (1-step, PEG, CDC method, *P* < 0.01; 2-step, PEG, CDC method, *P* < 0.005 [in WWTP-B]) (Fig. 5). As the viral dynamics and partitioning in wastewater are rather uncertain, the parallel detection of RNA in solid and liquid fractions would be ideal for sewage SARS-CoV-2 monitoring until a clear trend can be observed. For retrospective analysis, the storage of fractions reduced the concentration of SARS-CoV-2 RNA, but this can be corrected by normalization with the PMMoV RNA level (43). Alternatively, the purified RNA or cDNA samples from both solid and liquid fractions can be stored for future analyses.

Similar to the results of a previous report (31), the CDC N1/N2 duplex assay exhibited a higher rate of detection of SARS-CoV-2 RNA than the NIID N2 assay (Fig. 3 and Table 2). In the RT-qPCR assays, both the 1-step and 2-step methods exhibited equivalent performances (Fig. 3 and Table 2). While the 1-step RT-qPCR assay is time-effective, cDNA products after the RT reaction in a 2-step assay can be used for the detection of other RNA viruses such as norovirus, saving original RNA samples (44–46).

The differences in the stabilities and dynamics of SARS-CoV-2 detection between different WWTPs have been discussed previously (33, 47). In the present study, the rate of detection of SARS-CoV-2 RNA in samples derived from WWTP-B was higher than that in samples from WWTP-A, even when the case numbers were similar (Fig. 2). Fewer inhabitants live in the catchment area of WWTP-A than in that of WWTP-B (Table 1). The area of WWTP-B includes a large commercial area, which has several business establishments (Table 1). These factors may have contributed to the higher PMMoV RNA concentrations in the excreta in WWTP-B samples than in WWTP-A samples. Consistent with the results of previous studies (30, 31), the concentration of PMMoV RNA in the solid fraction was approximately 1 log_10_ unit lower than that in the liquid fraction, but it remained abundant and stable. We used PMMoV as an internal control without a spike procedure as it can be reliably detected in both the liquid and solid fractions of wastewater. Several studies have reported that the PMMoV copy numbers can be used to normalize the SARS-CoV-2 copy numbers (28, 48). However, as PMMoV is a nonenveloped virus, unlike SARS-CoV-2, the results of the quantification were not used for normalization or the calculation of the virus recovery rate but were used to check the substantial loss of SARS-CoV-2 detection in this study. Consistent with the findings of a previous report (38), no seasonal differences were observed in PMMoV detection during the entire monitoring period in both WWTPs (data not shown). The PMMoV RNA level in the wastewater of a community is associated with the consumption of food that contains peppers (37). As the PMMoV level in wastewater depends on eating habits in the surveillance area, preliminary monitoring is important before the implementation of an ES program.

Some samples in both WWTPs showed low concentrations of SARS-CoV-2 RNA that were below the limit of quantification (LOQ) (Fig. 3). They were detected in technical duplicates of RT-qPCR. The concentrations below the LOQ were also incorporated into the analyses in the present study. Some previous reports and guidelines used RNA concentrations that were one-half the corresponding LOQ (49–51).

In summary, the polio ES system successfully monitored the prevalence of enterovirus and SARS-CoV-2 for a long period. Recently, vaccine-derived polioviruses were detected from wastewater in the United Kingdom and the United States (52), highlighting the importance of polio ES even in polio-free countries. While wastewater samples are collected on a monthly basis for polio ES and enterovirus monitoring in Japan, the sampling frequency for SARS-CoV-2 in the present study was once per week (period 1) or once per 2 weeks (period 2). The results demonstrated a significant correlation between SARS-CoV-2 RNA concentrations in wastewater and the numbers of new COVID-19 cases. This suggests that the existing ES program, with sampling once every 1 to 2 weeks, can be used as a supplementary system for COVID-19 CS. Although more frequent sampling can improve the evaluation of trends of new cases in a community (50), it is also suggested that a novel sampling program should be aligned with the capacities of the existing ES system (22). Unlike the polio ES system, standard methods for SARS-CoV-2 ES are not available. Continuous improvement of an efficient method and system for ES implementation with a view toward cost-effectiveness is necessary for the post-COVID-19 era.

## MATERIALS AND METHODS

### Wastewater sampling for routine polio ES and COVID-19 response.

Influent wastewater samples in this study were collected from WWTPs located in the northeastern region of Japan. WWTP-A has the capacity to serve a population of 200,000 inhabitants, and WWTP-B can support 500,000 individuals (Table 1). For routine polio ES, grab influent wastewater samples (500 mL) were collected monthly from WWTP-A between January 2019 and December 2021 (*n* = 36). In response to the COVID-19 pandemic, additional wastewater samples (400 mL) were collected from both WWTPs (WWTP-A, *n* = 43; WWTP-B, *n* = 38) for SARS-CoV-2 monitoring from August 2020. There were two different sampling periods. Period 1 (August 2020 to March 2021 at WWTP-A and October 2020 to March 2021 at WWTP-B) had a weekly sampling frequency, and all samples were analyzed in the same laboratory. Period 2 (April 2021 to November 2021 at both WWTPs) included sampling every 2 weeks. A private laboratory, Shimadzu Techno-Research (Kyoto, Japan), analyzed the samples collected during period 2; this was done after conducting a technology transfer of the detection methods, including virus recovery methods and RT-qPCR assays. Samples for polio ES (500 mL) and SARS-CoV-2 monitoring (400 mL) were obtained separately. All samples were collected in sterile plastic bottles and immediately transported under refrigeration to the laboratories. The samples were frozen at −80°C until analysis, which was conducted within 7 days after collection.

### Enterovirus isolation from wastewater.

Wastewater samples (450 mL) were centrifuged at 2,900 × *g* for 60 min at 4°C. The resulting supernatant was used for PEG precipitation by the addition of PEG 8000 (final concentration, 12%) and NaCl (final concentration, 1 M), incubated overnight with gentle agitation at 4°C, and centrifuged at 11,900 × *g* for 20 min. Thereafter, the PEG precipitate was resuspended in 4.5 mL viral transport medium (VTM) (Eagle’s minimum essential medium [E-MEM] supplemented with 3.6% l-glutamine, 10% bovine serum albumin, penicillin-streptomycin-nystatin, and 7.5% sodium bicarbonate). The virus suspension was filtered through a 0.45-μm MF-Millipore filter (Merck, Tokyo, Japan). Thus, the wastewater sample was concentrated from 450 mL to 4.5 mL (100-fold concentrated). Virus isolation was performed using the RD-A, A549, Vero E6, LLC-MK2, and L20B cell lines (11–14, 40, 53). The cells were grown in E-MEM containing 10% fetal bovine serum (FBS) and penicillin-streptomycin. For virus isolation, cells were cultured with 2% FBS–MEM in 48-well plates; 50 μL of concentrated wastewater diluted with 50 μL of VTM (therefore, 50-fold-concentrated wastewater) was inoculated into each well containing the four cell lines. For each concentrated sample, 10 wells for each cell line were inoculated. Thus, a total of 40 wells were inoculated for 1 wastewater sample per month (1,440 wells for 3 years). Inoculated cells were observed daily (weekdays) under a microscope (CK2; Olympus, Tokyo, Japan) to detect the appearance of CPEs. After 7 days of observation, 100 μL of the supernatant from the CPE-negative culture was transferred for a second passage for an additional 14 days of observation. When CPEs appeared, isolates were harvested and reinoculated onto L20B cells for poliovirus detection. Poliovirus was not detected during this 3-year-long ES.

### Molecular typing of enterovirus by RT-PCR.

Viral RNAs were extracted from isolates using the QIAamp viral RNA minikit (Qiagen, Valencia, CA, USA) and analyzed using RT-PCR assays. Primers used in this study are listed in Table 3. Enterovirus typing was conducted using an enterovirus RT-PCR method modified from that described previously by Ishiko et al. (54). Reaction mixtures were prepared with a PrimeScript II high-fidelity one-step RT-PCR kit (TaKaRa Bio, Kusatsu, Japan) and primers EVP4mod and NR2C. The thermal cycling conditions for this enterovirus 1-step RT-PCR assay were “touchdown PCR,” as follows: an initial incubation step at 45°C for 10 min and an initial denaturation step at 94°C for 2 min followed by 5 cycles of denaturation at 98°C for 10 s, primer annealing at 65°C to 61°C (decreasing every cycle) for 15 s, and extension at 68°C for 60 s and then 40 cycles of denaturation at 98°C for 10 s, primer annealing at 60°C for 15 s, and extension at 68°C for 60 s. PCR products were analyzed by Sanger sequencing using the primers AN88 and AN89 (55). Enterovirus serotypes of the isolates were identified as having >75% similarity with the nucleotide sequences of the prototype enterovirus in the VP1 region and were determined using the Enterovirus Genotyping Tool version 0.1 (56). The PCR and RT-PCR assays for adenovirus and reovirus were previously described (57, 58) (primers are listed in Table 3), and the serotype was not determined for these virus species.

**TABLE 3:** Primers and probes used in this study

Assay and primer/probe function	Primer/probe name	Sequence (5′–3′)^*a*^	Reference(s)
Enterovirus			
Forward primer	EVP4mod	CGASTACTTTGGGWRWCCGTGTTTC	This study
Reverse primer	NR2C	TCAATACGGYRTTTGSWCTTGAACTG	
Forward primer	AN89	CCAGCACTGACAGCAGYNGARAYNGG	55
Reverse primer	AN88	TACTGGACCACCTGGNGGNAYRWACAT	
Adenovirus			
Forward primer	AdnU-S′2	TTCCCCATGGCNCACAAYAC	58
Reverse primer	AdnU-A2	TGCCKRCTCATRGGCTGRAAGTT	
Reovirus			
Forward primer	REOL3F	CAGTCGACACATTTGTGGTC	57
Reverse primer	REOL3R	GCGTACTGACGTGGATCATA	
NIID N2			
Forward primer	NIID_2019-nCoV_N_F2	AAATTTTGGGGACCAGGAAC	59
Reverse primer	NIID_2019-nCoV_N_R2	TGGCAGCTGTGTAGGTCAAC	
TaqMan probe	NIID_2019-nCoV_N_P2	FAM-ATGTCGCGCATTGGCATGGA-BHQ1	
CDC N1/N2			
Forward primer	2019-nCoV_N1-F	GACCCCAAAATCAGCGAAAT	60
Reverse primer	2019-nCoV_N1-R	TCTGGTTACTGCCAGTTGAATCTG	
TaqMan probe	2019-nCoV_N1-P	Cy5-ACCCCGCATTACGTTTGGTGGACC-BHQ1	
Forward primer	2019-nCoV_N2-F	TTACAAACATTGGCCGCAAA	60
Reverse primer	2019-nCoV_N2-R	GCGCGACATTCCGAAGAA	
TaqMan probe	2019-nCoV_N2-P	Cy5-ACAATTTGCCCCCAGCGCTTCAG-BHQ1	
PMMoV			
Forward primer	PMMV-FP1-rev	GAGTGGTTTGACCTTAACGTTTGA	36, 38
Reverse primer	PMMV-RP1	TTGTCGGTTGCAATGCAAGT	
TaqMan probe	PMMV-Probe1	FAM-CCTACCGAAGCAAATG-MGB-NFQ	

a FAM, 6-carboxyfluorescein; BHQ1, black hole quencher 1.

### Methods for SARS-CoV-2 RNA recovery from wastewater.

SARS-CoV-2 was recovered from wastewater samples using previously described methods (31). Briefly, the liquid and solid fractions were separated from the wastewater samples (500 mL for WWTP-A and 400 mL for WWTP-B) via centrifugation at 2,900 × *g* for 30 min. Subsequently, the liquid fraction (450 mL for WWTP-A and 360 mL for WWTP-B) was used for PEG precipitation; PEG 8000 (final concentration, 12%) and NaCl (final concentration, 1 M) were added to the supernatant. After incubation at 4°C overnight with gentle agitation and centrifugation at 11,900 × *g* for 20 min, the PEG precipitate was resuspended in VTM (4.5 mL for WWTP-A and 3.6 mL for WWTP-B). Using 1 mL of this solution, RNA was extracted with a QIAamp UltraSens virus kit (Qiagen) according to the manufacturer’s instructions. The solid fraction (4.5 mL for WWTP-A and 3.6 mL for WWTP-B) was obtained after the initial centrifugation step, from which RNA extraction was performed using the RNeasy PowerSoil total RNA kit (Qiagen) according to the manufacturer’s instructions.

### RT-qPCR assay for SARS-CoV-2.

SARS-CoV-2 RNA was quantified using two RT-qPCR assays, namely, the NIID 2019 novel coronavirus (2019-nCoV) N (NIID N2) assay (59) and a duplex assay of CDC 2019-nCoV N1 and CDC 2019-nCoV N2 (CDC N1/N2) (60). The sequences of the primers and probes used in these assays are listed in Table 3. For the 2-step RT-qPCR assay, extracted RNAs were reverse transcribed into cDNAs using PrimeScript RT master mix (Perfect real time) according to the manufacturer’s instructions. Reaction mixtures were prepared using one-step PrimeScript III RT-qPCR mix with uracil DNA glycosylase (UNG) (TaKaRa Bio) and primer/probe N2 (2019-nCoV) (TaKaRa Bio) for the NIID N2 assay and the SARS-CoV-2 RT-qPCR direct-detection kit (TaKaRa Bio) for the CDC N1/N2 assay. Thermal cycling was performed using QuantStudio 5 (Thermo Fisher Scientific, Waltham, MA, USA), and the thermal cycling conditions for RT-qPCR assays were as follows: initial incubation at 25°C for 10 min and 52°C for 5 min and then 45 cycles of denaturation at 95°C for 10 s and primer annealing and extension at 60°C for 30 s for the NIID N2 assay and initial incubation at 52°C for 5 min and then 45 cycles of denaturation at 95°C for 10 s and primer annealing and extension at 60°C for 30 s for the CDC N1/N2 assay.

### Quality control for SARS-CoV-2 RT-qPCR.

PMMoV RNA in wastewater samples was quantified as an internal control for viral RNA recovery and the effect of PCR-inhibitory substances via RT-qPCR using one-step PrimeScript III RT-qPCR mix with UNG (TaKaRa Bio) (31, 39). The primer and probe sequences used in this assay are listed in Table 3 (36, 38). The thermal cycling conditions for the PMMoV RT-qPCR assay included an initial incubation step at 25°C 10 min and 52°C for 5 min followed by 45 cycles of denaturation at 95°C for 10 s and then primer annealing and extension at 60°C for 30 s. In this study, values of >10^3^ copies/reaction were acceptable for the PMMoV RNA level, which is the equivalent of 10^5^ copies/L in the solid fraction of wastewater (31). The RT-qPCR protocols were performed according to guidelines for the minimum information for publication of quantitative real-time PCR experiments (61). All RT-qPCR assays for SARS-CoV-2 and PMMoV were performed in duplicate and included both negative and positive standard controls. To obtain standard curves for both assays, 10-fold dilution series of standard RNA controls were prepared. These included 5 × 10^0^ to 5 × 10^3^ and 1 × 10^2^ to 1 × 10^5^ gene copies/reaction for SARS-CoV-2 and PMMoV, respectively. The assay limit of quantification for SARS-CoV-2 was set at 5 gene copies/reaction as the lowest standard control. This was equivalent to different copy numbers in the solid-based and PEG precipitation methods. In WWTP-A wastewater, this value was equivalent to 200 copies/L and 600 copies/L, respectively, whereas in wastewater from WWTP-B, it was equivalent to 250 copies/L and 600 copies/L, respectively. Reaction mixtures containing fewer than 4 gene copies/reaction were occasionally amplified in only one of the duplicates, which were regarded as negative. To avoid contamination, RNA extraction and RT-qPCR preparation were performed in separate laboratory rooms, and the RT-qPCR mixtures were prepared on a clean bench, except for the addition of the template.

### Statistical analysis.

Spearman’s rank correlation test to analyze the correlation between the sewage SARS-CoV-2 RNA concentrations and the number of new COVID-19 cases and McNemar’s test to compare the rates of detection by each method were performed using GraphPad Prism 8 and the GraphPad website (GraphPad Software, San Diego, CA, USA), respectively. Significance was set at a *P* value of <0.05.

### Data availability.

The nucleotide sequences determined in this study were deposited in the GenBank/EMBL/DDBJ database under accession numbers LC734976 to LC735014.
