# Supplementary material for: Monitoring Enteroviruses and SARS-CoV-2 in Wastewater Using the Polio Environmental Surveillance System in Japan
Source: Appl Environ Microbiol. 2023 Mar 28;89(4):e01853-22. doi: 10.1128/aem.01853-22 (PMC10132113; doi:10.1128/aem.01853-22)
Supplement: Supplemental file 1 — Supplemental material. Download aem.01853-22-s0001.pdf, PDF file, 0.08 MB [file aem.01853-22-s0001.pdf]

Figure S1

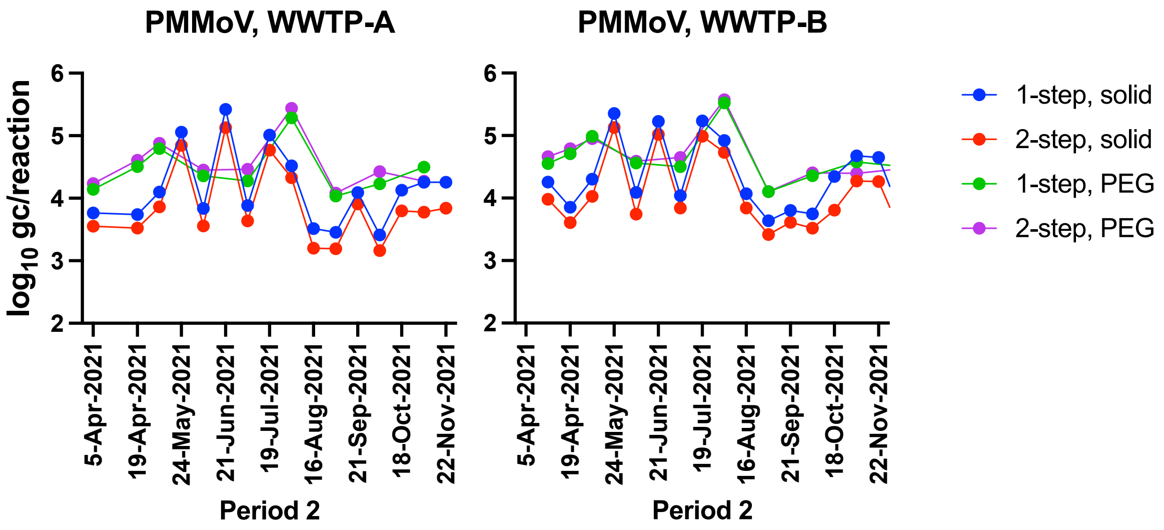

Figure S1 Summary of PMMoV RNA concentrations in the wastewater samples obtained from WWTP-A and WWTP-B during the Period 2. Data on RNA concentrations per reaction are presented separately based on the virus recovery method (solid-based and PEG precipitation) and the 1-step and 2-step of the RT-qPCR procedure.
